# Assessment of lower extremity muscle mass, muscle strength, and exercise therapy in elderly patients with diabetes mellitus

**DOI:** 10.1186/s12199-018-0710-7

**Published:** 2018-05-17

**Authors:** Takuo Nomura, Toshihiro Kawae, Hiroaki Kataoka, Yukio Ikeda

**Affiliations:** 1 Department of Rehabilitation Sciences, Kansai University of Welfare Sciences, Kashiwara city, Osaka 582-0026 Japan; 2 Division of Rehabilitation, Hiroshima University Hospital, Hiroshima, Hiroshima 734-8551 Japan; 3 Rehabilitation Center, KKR Takamatsu Hospital, Takamatsu, Kagawa 760-0018 Japan; 4 Diabetes Center, Kochi Memorial Hospital, Kochi, Kochi 780-0824 Japan

**Keywords:** Diabetes, Elderly, Muscle, Strength, Aging, Sarcopenia, Locomotive syndrome (locomo), Dynamometer, Exercise, Physical therapy

## Abstract

The increase in the proportion of elderly people in the population is one of the most remarkable sociodemographic phenomena of the twenty-first century. The number of patients with diabetes is also increasing worldwide with this demographic change. Given these facts, consideration of the problems the general elderly population is facing in the management of diabetes is essential. In this review article, we focus on sarcopenia, which is the decrease in lower extremity muscle mass and muscle strength accompanying aging, describe the relationship between sarcopenia and diabetes, and highlight the specific factors through which diabetes contributes to loss of muscle strength. The quantitative methods for evaluating lower extremity muscle strength will also be described. These methods hold the key to assessing the effectiveness of exercise therapy and optimizing the assessment of the degree of autonomy in the activities of daily living. Exercise is one of the basic treatments for type 2 diabetes and may also prevent and improve sarcopenia. This review discusses the aspects common to the two health conditions and elucidates the effectiveness and necessity of exercise as a preventive measure against diabetes among the elderly.

## Background

### Diabetes and aging

Currently, there are 425 million people worldwide with diabetes, and 158.8 million people with diabetes live in the Western Pacific Region. This number is the highest among the International Diabetes Federation regions [1]. Moreover, the number of patients with diabetes is increasing in Japan. The National Health and Nutrition Examination Survey issued in September 2017 estimated that there are 20 million diabetic and prediabetic patients, including 10 million diabetic patients in Japan [2]. While the total population of Japan is decreasing, the number of people aged ≥ 65 years is expected to continue rising after reaching a peak in 2042 [3], thereby further increasing the number of elderly patients. It is an acknowledged fact that the proportion of elderly diabetic patients will continue to grow. Therefore, the discovery of more effective measures to prevent diabetes is essential.

### Sarcopenia and locomo

One health problem that the general elderly population faces today is locomotive syndrome (locomo), whereby locomotive function declines due to motor dysfunction [4]. Sarcopenia is the degenerative loss of muscle mass and strength associated with aging [5], and it is significantly associated with locomo [6]. Yamada et al. reported that the prevalence rate of sarcopenia, determined using the European Working Group on Sarcopenia in Older People-suggested algorithm, in Japanese men and women aged 65 to 89 years was 21.8 and 22.1%, respectively [7]. Lower extremity muscle strength (LEMS) is closely related to locomotive function, and a recent study has revealed an association between loss of muscle strength and other motor dysfunctions and diabetes [8]. If medical staff only focus on the risk of LEMS deterioration in diabetic patients, other important factors may be overlooked, such as control of blood glucose levels, serum lipids, and blood pressure. In other words, diabetes treatment must be balanced and include all of these important assessments. Narrowing down the characteristics of diabetes that are associated with LEMS decline is important, but this has not been fully elucidated. Furthermore, the clinical efficacy of quantitative LEMS evaluation methods remains unclear, despite the established importance of focusing on LEMS decline in diabetic patients.

### Exercise therapy

Continuous practice of aerobic exercise improves blood glucose control, insulin resistance, fat metabolism, and cardiorespiratory function in patients with type 2 diabetes [9–11]. Even better results can be expected by combining aerobic exercise with resistance exercise [12]; thus, the combination of aerobic and resistance exercises is well established as a fundamental treatment for type 2 diabetes [13]. Although previous studies have shown nutrition, exercise, or their combination as effective methods for preventing or improving sarcopenia, further ongoing investigation is needed. For example, with regard to exercise, its effects on muscle mass have been inconsistent, and no consensus has been reached on any existing published guideline. However, resistance exercise has been reported as a potential effective means of improving muscle strength and physical function [14]. In the elderly in particular, exercise is essential for preventing and managing sarcopenia, given that it counteracts the decline in muscle strength associated with both aging and diabetes. The common aspects of exercise as a means of treating diabetes and as a means of preventing and managing sarcopenia are yet to be elucidated.

### Research questions

The research questions for this review article were the following:

1. When focusing on LEMS, what characteristics of diabetes should we examine in elderly patients?
2. Which quantitative methods for evaluating LEMS are the most effective in any field of diabetes care?
3. What are the optimal methods of exercise therapy for diabetes and for the prevention and management of sarcopenia in elderly diabetic patients?

## Muscle mass and diabetes

### Aging and muscle mass

In general, muscle mass starts to decline around the age of 40 years. In the years between the ages of 40–44 and 75–79, men and women lose 10.8 and 6.4% of muscle mass, respectively [15]. Sarcopenia has been reported to be an independent risk factor for diabetes [16, 17]. It correlates with the presence or absence of diabetic retinopathy as a characteristic complication of diabetes and its severity [18], and it is associated with the presence or absence of mild cognitive impairment [19]. The skeletal muscles require insulin for glucose uptake in peripheral tissues to be used as energy or stored in the form of glycogen. Reduction of skeletal muscle mass due to sarcopenia lowers glucose metabolism by insulin, leading to insulin resistance, thus making sarcopenia a factor contributing to the onset or exacerbation of diabetes [20]. Elderly diabetic patients have lower muscle mass and strength compared to nondiabetic elderly of the same age [21]. Therefore, the treatment of sarcopenia can play an important role in the overall treatment of diabetes in elderly patients affected by both diseases.

### Bodyweight and muscle mass

The Health, Aging, and Body Composition (Health ABC) study comparing 485 type 2 diabetic and 2134 nondiabetic participants in their 70s revealed that diabetic patients of both sexes had significantly higher leg muscle mass than nondiabetic participants (men, 9.1 ± 1.4 vs. 8.7 ± 1.3 kg; women, 7.0 ± 1.2 vs. 6.3 ± 1.2 kg) because they had bigger body size [22]. Although no difference was observed in the height of the Health ABC study subjects, diabetics weighed significantly more than nondiabetics (men, 85.3 ± 13.8 vs. 80.3 ± 12.6 kg; women, 76.9 ± 14.1 vs. 69.2 ± 14.1 kg), suggesting the possibility that natural body type may directly explain the difference in muscle mass [22]. More severe forms of diabetes could be a factor contributing to the exacerbation of sarcopenia, but when considering elderly diabetic patients as a whole, type 2 diabetics may not necessarily have lower muscle mass compared to nondiabetics.

The mean body mass index (BMI) of Japanese males and females aged 15 years and older in 2015 was 22.9 kg/m^2^, according to the National Health and Nutrition Survey [23]. The mean BMI of type 2 diabetes patients in the same year, as published by the Japan Diabetes Clinical Data Management Study Group, was 24.7 kg/m^2^, nearly 2 kg/m^2^ higher than the mean BMI of the general Japanese population, and there has been an increasing trend each year [24]. These findings suggest that contemporary Japanese type 2 diabetic patients weigh more than nondiabetics. Studies that match nondiabetics to diabetics by bodyweight therefore may not capture the general characteristics of diabetes. Our previous study comparing nondiabetics and type 2 diabetic patients aged 40–64 years revealed that the BMI of diabetic patients was significantly higher than that of nondiabetics, but no significant difference was observed in skeletal muscle mass [25]. In a cross-sectional observational study, muscle mass in type 2 diabetic patients was affected by the disease state of diabetes or the presence or absence of complications; thus, caution should be applied when interpreting results of comparative analyses.

### Inflammation and muscle mass

Compared to nondiabetics, elderly type 2 diabetic patients have higher levels of C-reactive protein, a marker for inflammation, and higher levels of interleukin-6 (IL-6), which has catabolic effects on muscles [26]. The Health ABC cohort study reported that the decrease in skeletal muscle mass was significantly higher in diabetic patients than in nondiabetics over a 3-year period [27]. Results of longitudinal observations indicate that diabetes is a factor that accelerates the muscle mass decrease associated with aging. The potential mechanism is increased levels of inflammatory cytokines in patients with diabetes. Systemic inflammatory cytokines, such as tumor necrosis factor-alpha and IL-6, may have detrimental effects on muscle mass in older adults [27, 28].

## Muscle strength and diabetes

### Diabetic neuropathy and LEMS

The mechanisms underlying motor dysfunction in diabetic patients are complex. Abnormal mitochondrial function and free fatty acid metabolism, as well as an inadequate increase in the microvascular blood supply during exercise, are also likely to affect muscle function [29, 30]. Although there are some differences according to body part, muscle mass correlates with muscle strength; therefore, reduction in muscle mass decreases muscle strength. Furthermore, diabetic neuropathy (DN), a complication that characterizes diabetes, also decreases muscle strength, while more severe degrees of DN further exacerbate this decline [31]. One of the common forms of DN is diabetic polyneuropathy (DPN), and symptoms originate in the lower limb extremities and move upward, eventually manifesting in the upper extremities as well [32]. Andersen et al. compared 36 nondiabetics to 36 type 2 diabetic patients (mean age, 58.5 years; mean disease duration, 11 years) and found that type 2 diabetic patients had lower ankle dorsiflexion force (by 17%, significant) and knee extension force (KEF, by 7%, not significant) than nondiabetics [31]. Further, an examination of diabetic patients alone has revealed larger decreases in KEF according to the presence and degree of DPN.

Some studies have examined DPN complications and muscle strength in small samples of diabetic patients [31, 33], whereas other large-sample studies have examined elderly patients without consideration of DPN complications [22, 27]; however, there are no existing studies that have examined DPN complications in a sample with a wide age range. We conducted a multicenter study on KEF in 1442 type 2 diabetic patients with a wide age range (Multicenter Survey of the Isometric Lower Extremity Strength in Type 2 Diabetes (MUSCLE-std) study) [34]. A univariate analysis comparing diabetic patients by age group (three groups: 30–49, 50–69, and 70–87 years) revealed that in men and women aged 30–49 years, the patients with a complication of DPN had somewhat lower KEF than patients without DPN, but the difference was not significant [35]. However, in this study, both men and women with DPN aged 50–69 and 70–87 years showed significantly lower KEF (11.0–12.9 and 11.9–16.6%, respectively) compared to those without DPN, revealing that DPN was a significant independent explanatory variable of KEF. Moreover, the MUSCLE-std study suggested that maintaining KEF was also effective in maintaining exercise habits [36].

### Bodyweight and LEMS

To evaluate LEMS, the bodyweight ratio is often used as values are normalized by weight to correct for differences in body types. When the muscle strength-to-bodyweight ratio is used to compare two groups with a difference in body type, bodyweight can have a large impact on muscle strength; hence, this ratio must be used with caution (e.g., when you compare muscle strength between groups with a significant difference in bodyweight). Diabetics weighed significantly more than nondiabetics in the Health ABC study, and this study divided muscle strength by muscle mass to normalize values [22, 27]. Rather than dividing muscle strength by weight, the ratio of muscle force to muscle mass may better represent muscle quality and may also be a better index for evaluating muscle strength in diabetic patients. However, its use is limited due to the difficulty in measuring muscle mass in some patients. Previous studies, including our own, have shown that KEF decreases more rapidly in type 2 diabetic patients compared to nondiabetics and that this loss may range between 10 and 20%. Diabetic patients with DPN have significantly lower LEMS compared to those without DPN, but this relationship is more prominent in older patients.

### Motor function and LEMS

Among LEMS, KEF is closely associated with basic activities of daily living (ADLs), such as standing and walking. The National Health and Nutrition Examination Survey (1999–2002) on diabetic patients aged 50 years and older demonstrated a significantly negative correlation between diabetes duration and KEF corrected by age [37]. This report suggests that diabetic patients walk at significantly slower speeds than nondiabetics (0.96 ± 0.02 vs. 1.08 ± 0.01 m/s). A study examining the relationship between muscle strength and ADL in community-dwelling elderly individuals showed that the KEF required for ADL independently was 2.8 N/kg (muscle strength/bodyweight) [38]. A study on healthy elderly people aged 74 ± 7 years reported that a decline in KEF increases the risk of falls [39]. In diabetic patients, complications with DPN not only cause motor dysfunction but also impair sensory function, which increases the risk of falls due to loss of balance [40]. Focusing on KEF is an effective way to determine the degree of autonomy in walking or ADL as well as evaluating the effectiveness of exercise therapy. Particularly in elderly diabetic patients, KEF is an essential index when implementing exercise therapy programs aimed at preventing the need for long-term care.

## Evaluation of muscle mass and muscle strength

### Evaluation of muscle mass

Muscle mass is used in diagnosing sarcopenia. Computed tomography (CT) scanning and magnetic resonance imaging (MRI) permit accurate differentiation of the bone, fat, and lean body tissue and are gold standards for muscle mass evaluation [5]. However, these methods require large, expensive, and nonportable equipment, and therefore, there are limitations to their use in routine clinical practice. On the other hand, dual-energy X-ray absorptiometry (DXA) results in low radiation exposure among patients. The Health ABC study reported that participants were assessed using DXA and were classified as sarcopenic using two different approaches of adjusting lean mass to body size: appendicular lean mass divided by height squared and appendicular lean mass adjusted for height and body fat mass (residuals) [41]. In addition, the cutoff values of skeletal muscle mass index by DXA (appendicular skeletal muscle mass index (kg)/body height (m)^2^) were 7.23 kg/m^2^ for men and 5.67 kg/m^2^ for women. Unfortunately, DXA is also a nonportable equipment. Another method used is bioelectrical impedance analysis (BIA), which measures fat mass and lean body mass. Although the reliability of BIA is somewhat compromised in patients with a BMI of ≥ 35 kg/m^2^, it is a relatively inexpensive and portable alternative to DXA for accurate evaluation of body composition [42]. A working group in Asia reported diagnostic criteria for sarcopenia for Asians in 2015, and the cutoff values for muscle mass measured by BIA were reported [43]. The cutoff values for BIA were 7.0 and 5.7 kg/m^2^ for men and women, respectively. However, no standardized diagnostic criteria are available for sarcopenia, and results differ depending on the diagnostic criteria used.

### Evaluation of LEMS

The methods used to evaluate LEMS are broadly divided into manual muscle testing (MMT) [44] and machine method testing. The judgment is very subjective, as the intensity of the resistance force applied by hand by the tester in MMT grade 4 depends on the age and sex of the tester. Nevertheless, a large spread of muscle strength values can be measured using machine testing as well [45, 46]. To test for grade 5 MMT results, the tester must apply the maximum resistance by hand, but again, it is inevitable that there is a difference between the intensity of resistance force applied by a large man or a small woman [47, 48]. The machines used for measuring muscle strength include isokinetic dynamometers and handheld dynamometers (HHDs), which vary from expensive and large machines to relatively inexpensive and small devices. Muscle strength values obtained in the isokinetic mode by an isokinetic dynamometer are measured in terms of torque (units: Nm) under a set angular velocity. Meanwhile, the muscle strength values obtained with an HHD are in N units. When muscle strength is measured in kilogram-force (kgf), the conversion rate of 1 kgf = 9.8 N is used. In other words, an isokinetic dynamometer measures isokinetic muscle strength, while an HHD measures isometric muscle strength.

The reliability of muscle strength values measured using isokinetic dynamometers is reported to be high [49, 50], but this machine is large and expensive; thus, its wider application in clinical practice is limited. In contrast, HHDs are small, easily portable, and relatively inexpensive, but obtaining the same level of reliability as the isokinetic dynamometer is more difficult [51, 52]. Some studies have sought to improve the weaknesses of HHD. In one study, the subjects were instructed to maintain an upright posture with the hip and knee joints bent at 90° in an end-sitting position [53]. The HHD sensor pad was placed on the anterior face of the distal part of the lower leg of the subject, while the length of the stabilization belt was adjusted and tied to the posterior column supporting the testing board. During measurement, the tester supported the sensor pad lightly to prevent it from shifting. The intra-class correlation coefficients (ICCs) of measurements on the young participants (mean age 21.9 years) in that study were reported to be ≥ 0.9. On the other hand, the ICC of the same measurements taken on elderly persons (aged 65–79 years) was also reported to be ≥ 0.9 [54]. Furthermore, the correlation coefficient between isometric KEF measured with an HHD and a stabilization belt versus that measured with an isokinetic dynamometer was 0.75 [55]. As such, using a stabilization belt with an HHD makes it possible to achieve levels of validity and reliability equivalent to measuring muscle force with an isokinetic dynamometer.

## Exercise as a treatment for diabetes and for improvement and prevention of sarcopenia

### Exercise for diabetes

Exercise therapies for type 2 diabetes can be classified as either aerobic exercises (e.g., walking and bicycling) or resistance exercises (e.g., exercise using machines and elastic resistance bands). Balancing exercises are also important for preventing falls and accidents. Furthermore, unstructured physical activities are essential for reducing the number of consecutive sedentary hours, thereby reducing the total time spent seated.

The American Diabetes Association (ADA) recommends the following aerobic exercise regime for the general adult population with type 2 diabetes [56]. The recommended intensity is moderate to vigorous (subjectively experienced as “moderate” to “very hard”) and practiced at a frequency of 3–7 days a week (with no more than 2 consecutive days without exercise), totaling at least 150 min per week. Resistance exercises should be performed gradually, and the ADA recommends the following: exercise at moderate (e.g., 15 repetitions of an exercise that can be repeated no more than 15 times) to vigorous (e.g., 6–8 repetitions of an exercise that can be repeated no more than 6–8 times). The frequency is a minimum of 2 nonconsecutive days a week, but preferably 3, performing at least 8–10 exercises with completion of 1–3 sets of 10–15 repetitions to near fatigue per set on every exercise early in training. Flexibility training and balance training are recommended as follows: stretch to the point of tightness or slight discomfort. In each exercise performed, hold static or do dynamic stretch for 10–30 s, with 2–4 repetitions of each exercise and a frequency of ≥ 2–3 days a week.

The Japan Diabetes Society (JDS) recommends the following for aerobic exercises: moderate intensity at 50% of the maximum oxygen uptake (VO_2_ max) to maintain a heart rate of 100–120 beats/min for individuals aged 49 years and below and 100 beats/min for individuals aged 50 years and above, at a frequency of at least 3 nonconsecutive days per week, for 15–30 min per session [57]. Like the ADA, the JDS recommends resistance exercises as a treatment for diabetes, but does not give details on how they should be conducted.

### Exercise for sarcopenia

With regard to sarcopenia, there are no globally accepted guidelines in terms of effective exercise methods for its prevention and improvement. Law et al. reviewed the effectiveness of resistance exercises on sarcopenia and proposed methods of practicing resistance exercises [58]. The following regime was proposed: high intensity (≥ 80% of 1 repetition maximum (RM)) for 2–4 nonconsecutive days per week, working on a separate muscle group each day for 8–15 repetitions per session at 1 RM 80%, or 1–3 sets of 4–12 repetitions per session at 1 RM 90%.

### Exercise for diabetes and sarcopenia

Resistance exercises are recommended both for the treatment of type 2 diabetes and for the prevention and management of sarcopenia. There are no major differences between the intensity, frequency, number of repetitions, or sets per session of resistance exercises recommended by the ADA and those recommended for prevention and management of sarcopenia. However, strict blood pressure control is required according to the severity of the diabetic retinopathy or nephropathy [59]. High-intensity resistance exercise increases blood pressure; thus, it is crucial to make appropriate individual adjustments. Risks must be managed for diabetic patients with complications following tests for exercise loads, under the guidance and monitoring of exercise experts, such as a physical therapist.

On the other hand, adhering to exercise therapy is difficult for patients with diabetes [60, 61]. With regard to exercise therapy education, it is important to maintain the motivation of the patients to encourage participation in exercise therapies, to increase the frequency of guidance, and to provide a more detailed exercise prescription, in terms of frequency and duration [62]. Patient education using the transtheoretical model approach seems useful for exercise therapy in type 2 diabetes [63].

## Conclusions

The prevention of motor function decline is essential when considering the goals of care for elderly patients with diabetes [64]. Diabetes is a factor that exacerbates the decline of motor function associated with aging, and the presence of DPN, particularly severe DPN, further accelerates the progression of motor dysfunction. Quantitative evaluation of KEF using reproducible methods and its application to patient care is effective for the prevention and management of sarcopenia in elderly patients with diabetes. The HHD combined with a stabilization belt can evaluate LEMS in any field of diabetes care. The ratio of muscle force by HHD with a belt fixed to the muscle mass measured by BIA may better represent muscle quality and may also be a clinically better index for evaluating muscle strength in diabetic patients. Resistance exercises are commonly recommended for treating diabetes and for preventing and managing sarcopenia. Patients with diabetic complications must be monitored carefully if they undertake resistance exercise training. In addition, adherence to exercise programs is a major problem for diabetic patients. Future studies assessing the lifestyle intervention factors that influence adherence to exercise programs in diabetic patients are necessary.

## Abbreviations

ADA: American Diabetes Association
ADL: Activity of daily living
BIA: Bioelectrical impedance analysis
BMI: Body mass index
CT: Computed tomography
DN: Diabetic neuropathy
DPN: Diabetic polyneuropathy
DXA: Dual-energy X-ray absorptiometry
Health ABC study: Health, Aging, and Body Composition study
HHD: Handheld dynamometer
ICC: Intra-class correlation coefficient
IL-6: Interleukin-6
JDS: Japan Diabetes Society
KEF: Knee extension force
LEMS: Lower extremity muscle strength
Locomo: Locomotive syndrome
MMT: Manual muscle testing
MRI: Magnetic resonance imaging
MUSCLE-std study: Multicenter Survey of the Isometric Lower Extremity Strength in Type 2 Diabetes Study
RM: Repetition maximum
